# Assessment of Influences of Stenoses in Right Carotid Artery on Left Carotid Artery Using Wall Stress Marker

**DOI:** 10.1155/2017/2935195

**Published:** 2017-01-15

**Authors:** Arindam Bit, Dushali Ghagare, Albert A. Rizvanov, Himadri Chattopadhyay

**Affiliations:** ^1^Department of Biomedical Engineering, National Institute of Technology, Raipur, India; ^2^Kazan Federal University, Kazan, Russia; ^3^Department of Mechanical Engineering, Jadavpur University, Kolkata, India

## Abstract

*Purpose*. Atherosclerosis is a diseased condition of blood vessel. It causes partial blockage in lumen of vessel and affects hemodynamic of localized flowing blood. Complex geometries like region of bifurcation also affects hemodynamic to a larger extent. Complexity further increases in presence of stenoses at region of bifurcation. Such morphological change in vessel largely affects parent as well as corresponding sister and daughter vessels. In this paper, complexity in hemodynamic of blood in pair of carotid arteries (left and right carotid arteries) is evaluated in presence of stenoses at basilar segment of right artery in three-dimensional domain using reconstructed tomographic images of patient.* Methods*. Transient information of blood flow is obtained using four-dimensional phase-contrast MRI technique. Haematocrit component of blood at diseased condition is considered using Power Law and Quemada model. Numerical techniques are used to solve pressure-coupled governing equations of flowing blood.* Results*. Dysfunctions of endothelial cells near the wall are characterised by evaluating shear stress markers. Wall shear stress and its gradient based and harmonic based descriptors are calculated over complete geometry during one cardiac cycle.* Conclusion*. Internal branch of left carotid artery and external branch of right carotid artery are found prone to secondary stenoses in presence of primary stenoses at basilar segment of right carotid artery.

## 1. Introduction

Atherosclerosis is a vascular disease, and it is one of the major life-threatening factors in developed countries due to current lifestyle of their population. Thus, finding a solution to this mortality is factor of significant concern. While addressing the problem, many researchers had found existence of complex hemodynamic features in blood vessels with atherosclerosis. Partial blockage of blood vessels, known as stenoses, is one of the outcomes of atherosclerosis. Blood vessel containing bifurcation acts as flow-divider zone, being prone to stenoses. It modulates local hemodynamic structure of flowing blood, putting additional stress on inner lining of wall.

Drikakis et al. [1] considered simplest form of pulsating flow (sinusoidal) for characterising pattern of blood flow during reconstructive surgery. Blood was considered as non-Newtonian fluid in their study. Stenosed geometry was constructed as a function of upstream length in another study [2]. Stenosis was made independent of position in circulatory system. Jinyou and Yang [3] considered CT scan data for geometric-reconstruction of a healthy blood vessel. Input-velocity was considered as function of time. Morbiducci et al. [4] had calculated blood rheology marker for bulk flow in models of carotid bifurcation. Newtonian rheology was hold true for bulk flow metrics and found to influence wall shear stress at different levels of geometry. Physiological-relevant significant flow behaviour was also considered using simplified sinusoidal pulsating function [5]. Various opinions for modelling viscosity of blood within complex geometry were observed over time. Non-Newtonian viscosity models were also found effective for transient study of flow in complex geometries like arch of aorta and region of bifurcation [6, 7]. Flow disturbance in carotid artery containing stenoses was also evaluated [8]. Variations of carotid bifurcation of young and older population are also observed for access of risk of atherosclerosis [9]. A robust and objective technique was used to characterise three-dimensional geometry of bifurcation of carotid artery. Less interindividual variations in geometric parameters were observed in younger vessels than in older one. The study suggested availability of more complex interrelationship between vascular geometry, local hemodynamics, vascular ageing, and atherosclerosis. Large-scale simulation was done for human arterial-tree at healthy condition [10]. A number of investigators had considered bifurcated region of carotid artery for study of localized hemodynamic of blood [4]. Gallo et al. [11] had formulated helical-flow of blood in region of carotid bifurcation. This phenomenon was used as surrogate marker for prediction of disturbed shear. Helicity-based bulk flow descriptor was used to calculate the regions potential for exposure to disturbed shear.

Literature illustrates few investigations on study of hemodynamic in healthy carotid artery. However, complex flow structures were observed in all these studies. But assumptions like Newtonian nature of viscosity of blood and generic pulses of velocity-waves at inlet deviate actual conditions of the problem. In this paper, assumptions are refined to meet reality of the in vivo environmental condition. Blood is considered as non-Newtonian. Transient-flow information of blood flowing through stenosed artery (presence of 80% asymptomatic blockage in lumen of basilar segment of internal carotid artery of right carotid artery) of specific patient is used at inlet of the specified segmented flow section of the geometry. Simultaneously flow structures are investigated in both carotid arteries, with stenoses (diseased artery) in right carotid artery and without stenoses (healthy artery) in left carotid artery. Effects of change in velocity magnitude of flow rate resembling various competitive physiological conditions on flow structures are also evaluated in this study.

## 2. Methodology

This section includes brief description of geometry development, governing equations, boundary conditions, viscosity model, grid convergence test, and solution methodology.

**Figure 1:**
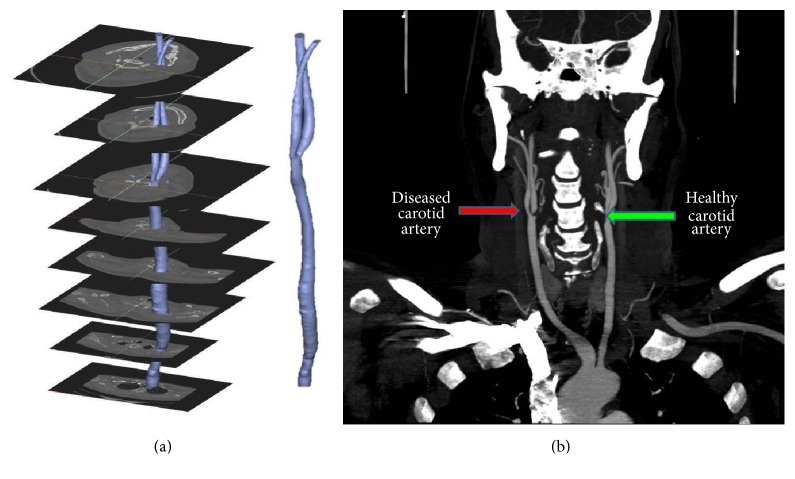
(a) 3D reconstruction of CAs from stack of CT scan slices (images); (b) front view of CT image showing RCA (diseased) and LCA (healthy).

Three-dimensional structures of left and right carotid arteries are reconstructed from stack of tomographic images (Somatom Definition AS 64 slices CT) of a patient suffering from carotid atherosclerosis at Sri Aurobindo Institute of Medical Sciences and Hospital, Indore, India. Reconstructed model of LCA is shown in Figure 1(a). Front view of CT scan image shows stenoses associated with region of bifurcation (at the onset of ICA and CA: 80% asymptomatic blockage in lumen of basilar segment of ICA) of RCA, as shown in Figure 1(b).

Semi-implicit methods are used in numerical domain for analyzing flow structures in the above-mentioned complex geometry. Finite element modelling of carotid artery is done by discretization of the geometry. Optimum mesh size is achieved after performing grid convergence test. Detail of grid convergence test is discussed later. Mesh statistics of final finite element model thus obtained are given in Table 1.

Compressibility of fluid due to localized deflections in pressure is neglected in order to simplify the problem. However, ratio of mean-deflection of vessel to mean-diameter of vessel is found negligible during normal metabolic activity. Also, during any hyperactive physiological state, brain sends an electropotential signal through efferent neurons and synapses of neurotransmitter for dilating smooth endothelial cells of blood vessel. The signal molecules (acetylcholine) released at neurovascular junction cause nearby epithelial cells to produce nitric oxide (NO), which then causes vascular dilation. This phenomenon eases the vessel to expand and accommodates increased blood flow. Response time of vascular dilation by this process is longer than rate-responsive cardiac cycle. Thus, vessel-wall can be treated as quasi-stationary for one cardiac cycle with respect to one complete cycle of its contraction-relaxation phase. Therefore, carotid artery can be treated as rigid during the entire transition of hemodynamic study. This makes the mass-conservation continuity equation independent of compressibility factor near wall. And it is represented by

(1) $$\begin{array}{c} \frac{1}{r}\frac{\partial }{\partial r}\left(\;rv\;\right)+\frac{\partial }{\partial x}\left(\;u\;\right)=0. \end{array}$$

In the above equation, *u* = *u*_*i*_, *i* ∈ 1,2, 3, corresponds to *x*-, *y*-, and *z*-axis, respectively. Such representation of *u* makes continuity equation independent of geometry of symmetry, particularly in postbifurcation region, because lumen of the stenosed artery is axially nonsymmetric but radially symmetric. Similarly, the conservation of momentum equation along axis of symmetry as well as along radial direction can be formulated as a function of a transient term, convective term, a negative pressure term, and diffusion term and is represented by (2) and (3).

Axial-momentum equation is
  (2) ∂u∂t+1r∂∂rruv+∂∂xu2=−1ρ∂p∂x+1r∂∂rμappρr∂u∂r+∂v∂x+∂∂x2μappρ∂u∂x.
*r*-Momentum equation is
  (3) ∂v∂t+1r∂∂rrv2+∂∂xuv=−1ρ∂p∂r+1r∂∂r2μappρr∂v∂r−2μappρvr2+∂∂xμappρ∂u∂r+∂v∂x.

**Figure 2:**
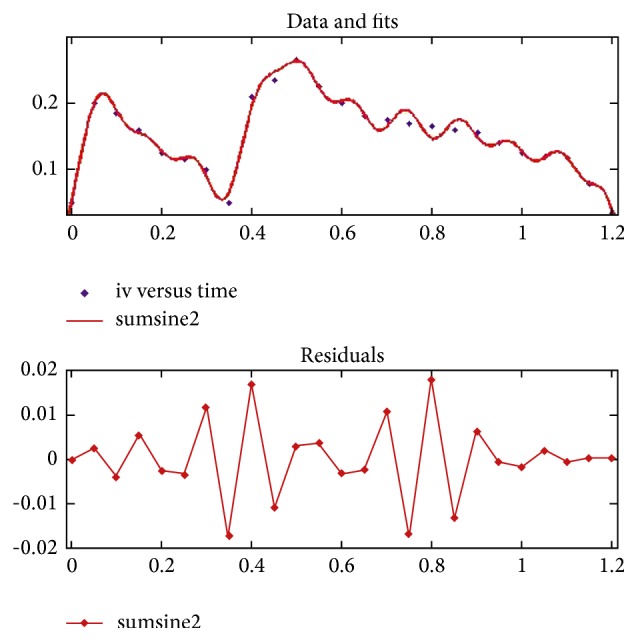
Inlet-velocity profile with residual error(s).

Arterial wall of stenosed-CA is realised as rigid. Rigidity of vessel is valid transiently with respect to one complete rate-responsive cardiac cycle. Therefore, for transient analysis, no-slip boundary condition is used at wall. Since whole system (stenosed blood vessel) is submerged in body fluid, therefore, zero gauge-pressure is considered at output end of both CAs (at posterior and anterior section of left and right carotid arteries). A unique inlet-velocity profile is obtained at the onset of left and right carotid artery from brachiocephalic region on the aortic arch. Differences in Womersley flow rate in individual vessels are merged to form a unique Womersley flow profile after applying highest time-resolution probe on the oblique plane confirming 94% similarity index in spatiotemporal transient response of blood entering in these two vessels, respectively. Also, since both LCA and RCA are originated from oblique plane overlapping intermediate distance, therefore, assumption of similar velocity profile holds true. Four-dimensional phase-contrast MRI technique is used to evaluate transient response of blood flowing from aorta to carotid artery. A time-averaging technique is used over five cardiac cycles to calculate mean-velocity profile at the onset of CAs. Velocity-waveform thus obtained (Figure 2) is represented by

(4) ut=0.1223−0.08081cos⁡ωt+0.05008sin⁡ωt+0.01045cos⁡2ωt+0.02759sin⁡2ωt+0.01747cos⁡3ωt+0.05915sin⁡3ωt+0.01034cos⁡4ωt+0.0504sin⁡4ωt+0.0002387cos⁡5ωt+0.01144sin⁡5ωt+0.01345cos⁡6ωt+0.01187sin⁡6ωt+0.002937cos⁡7ωt+0.01849sin⁡7ωt−0.003077cos⁡8ωt+0.009732sin⁡8ωt.

*ω* = 4.436 and values of coefficients *a*'s and *b*'s are given in Table 2.

Equation (4) is obtained by maintaining a goodness of fit with SSE = 0.003907, *R*^2^ = 0.9532, adjusted *R*^2^ = 0.8395, and *ε*_rms_ = 0.02362. Obtained profile is centreline-velocity profile. It is transformed into Womersley profile. “*h*” is considered as inlet diameter of respective values for both LCA and RCA. Let *X*[*i*, *j*] be a two-dimensional position-vector for centroid of individual discrete control-volume. A nondimensional representation of *y*-coordinate is made by using

(5) $$\begin{array}{c} y=2\frac{\left(x\left[i,j\right]-0.5h\right)}{h}. \end{array}$$

Equation (5) is used to evaluate Womersley profile using

(6) $$\begin{array}{c} v\left(\;y,t\;\right)=\left(\;1-y^{2}\;\right)v\left(\;t\;\right). \end{array}$$

Irrespective of equal velocity profile implemented at inlets of both CAs, Re varies in respective CAs due to difference in diameters of inlet of both vessels. Flow rate is made double and quadruple to evaluate effect of increased flow rate. Formulated Re are tabulated in Table 2.

Blood flowing through both CAs (smaller diameter vessels) exhibit nonlinear stress-strain relationship [12]. Thus, blood flowing in CAs is considered non-Newtonian in nature. During diseased condition, concentration of haematocrit in blood increases. It also makes blood non-Newtonian. Power Law model [13] is used to represent non-Newtonian behaviour. In this model, dynamic of haematocrit-concentration gradient and concentration gradient of total protein excluding albumin is taken into account. It is represented by system of the following equations:

(7) τ−=kγ−n−1γ−,μγ−∗=kU∞n−1ln−1γ−∗n−1.

For normal blood samples, parameters are *k* = 14.67 × 10^−3^ Pa s and *n* = 0.7755 [14]. If *n* > 1, the fluid is known as shear-thickening; otherwise, if *n* < 1, it is known as the shear thinning fluid.

Viscosity of concentrated disperse system (haematocrit in blood) was modelled by Quemada [15], which is also used here for comparative assessment with Power Law model. Quemada model is based on shear rate and concentration of haematocrit. System of equations for expressing shear stress and effective viscosity are described in the following equations:

(8) τ−=μF1−12k0+k∞γ−/γc1+γ−/γcφ−2γ−,μγ−∗=μF1−12k0+k∞γ−∗/γc∗1+γ−∗/γc∗φ−2,

where *γ*_*c*_^*∗*^ = *γ*_*c*_/(*U*_*∞*_/*l*) and the parameters are given as *μ*_*F*_ = 1.2 × 10^−3^ Pa·s, *φ* = 0.45, *γ*_*c*_ = 1.88 s^−1^, *k*_*∞*_ = 2.07, and *k*_0_ = 4.33.

SIMPLE-formulation [16] method is used to solve equations of fluid-mechanics. In order to achieve higher accuracy, convective terms are discretized using QUICK-scheme. Fixed time-step of size 0.0003 sec to 0.0001 sec is used with second-order accuracy in time-domain. Oscillatory problem of pressure is satisfactorily handled by using collocated-grid arrangement in momentum-interpolation method [17]. It involves appropriate interpolation of velocity-field at the interface of cell-faces. Interpolation of momentum is equivalent to evaluate continuity equation with an added fourth derivative of pressure. It does not alter the second-order accuracy of basic discretization process. The procedure was also successfully implemented in a previous work by Nandi and Chattopadhyay [18].

## 3. Results

Flow structure of blood racing to upper neck region and brain gets effectively influenced by the anomalies in right carotid artery. In this section, effective flow structures are observed in the diseased vessel (right carotid artery) by streamline velocity plot at regular time steps of a cardiac cycle and by observing contour of wall shear stress on the wall of the vessel. Also, an attempt is made to investigate the effect of stenoses in diseased vessel to the flow structures of blood in a connected healthy vessel (i.e., left carotid artery), in order to observe the alterations in phenomena of parallelism. Hemodynamics is also studied at higher velocities in order to investigate the risk of vessel rupture at higher rate of brain activity like mental-tension, brainstorming games, or accidental impact on the region of upper neck.

**Figure 3:**
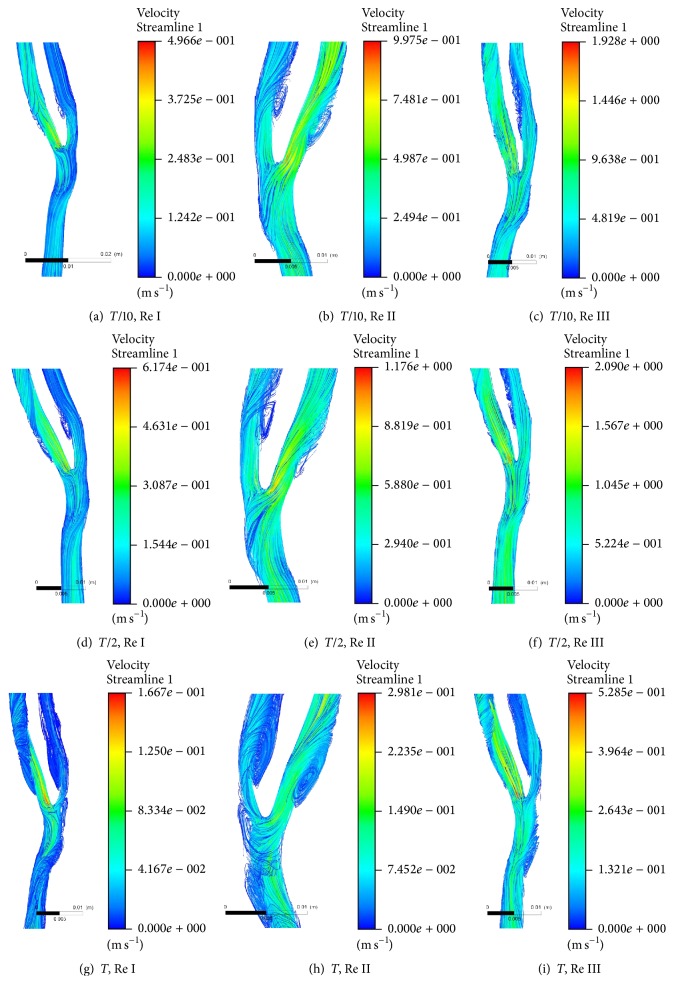
Streamline plot of velocity at bifurcated section of healthy carotid artery at* T* cycle at Re I (544), Re II (1088), and Re III (2176).

Figure 3 shows the variations of streamline of velocity plot at bifurcated section of healthy carotid artery at* T* cycle at different Re of 544, 1088, and 2176 while considering Power Law rheological model for realising viscosity of diseased state blood. Velocity streamline at* T*/10,* T*/2, and* T* is compared at flow rates Re I, II, and III, respectively. Maximum velocity of the fluid is observed at the region of bifurcation in all the cases. Magnitude of maximum velocity is found conserved with the increased flow rates at the above-mentioned time steps. However, it is found that vortices get attenuated with increased flow rate. Both internal and external arm of left carotid artery along with main branch of left carotid artery are found to have vortices whose dimensions get reduced on increased flow rate. However, vortices vanished from internal branch of left carotid artery at highest flow rate of Re III.

**Figure 4:**
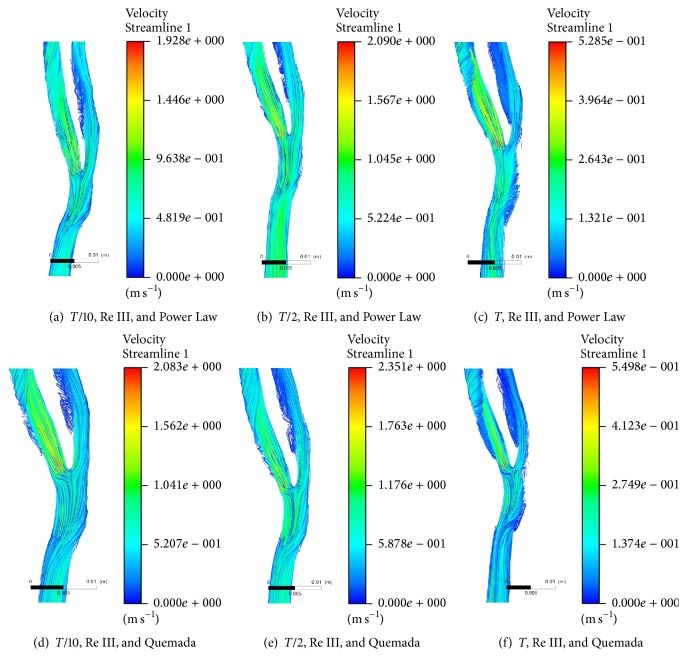
Streamline plot of velocity at bifurcated section of healthy carotid artery at Re 2176 while considering Power Law and Quemada rheological model.

Figure 4 shows a comparison of streamline of velocity plot at bifurcated section of healthy carotid artery at Re 2176 for Power Law and Quemada rheological models. Velocity streamlines at* T*/10,* T*/2, and* T* are compared at two different rheological models for blood as mentioned above. Flow structures of fluid flowing through the geometry are found almost similar in pattern at both viscosity models. Even distributions of vortices are also found similar in both the cases.

However, minute deviation in velocity magnitude is observed in between these two models. Quemada model exhibits a greater response to velocity magnitude of flowing fluid in comparison to Power Law model by a factor of 1.08. Quemada model is also found to respond to flow physics of higher shear rate in an improved manner than Power Law model.

**Figure 5:**
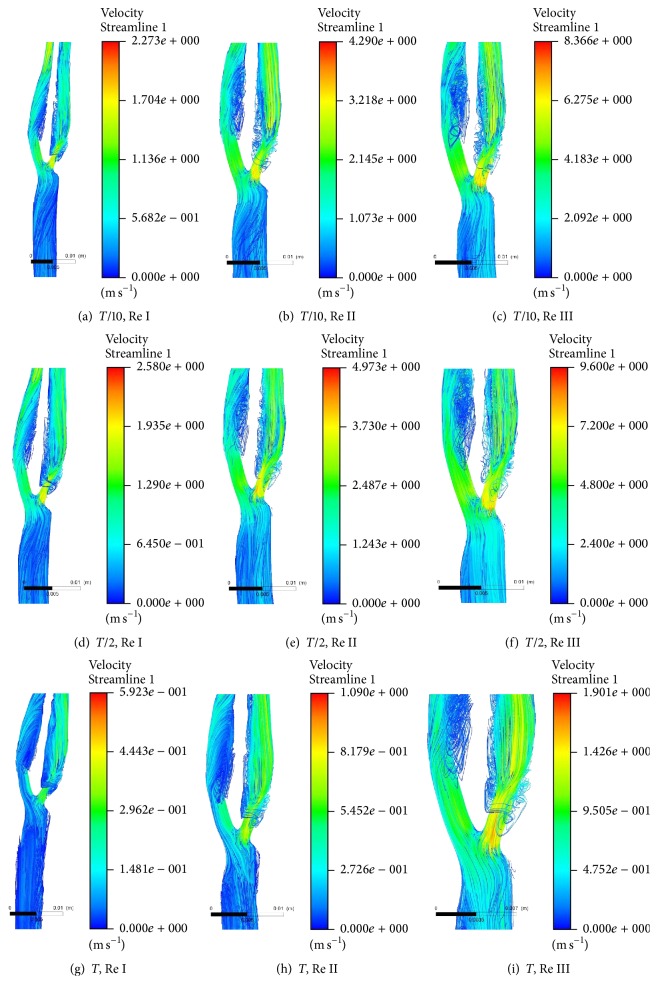
Streamline plot of velocity at bifurcated section of diseased carotid artery at* T* cycle at Re 954, 1908, and 3816.

Figure 5 shows the variation of streamlines of velocity plot at bifurcated section of diseased carotid artery at* T* cycle at different Re of 954, 1908, and 3816 while considering Power Law rheological model. Velocity streamlines at* T*/10,* T*/2, and* T* are compared at flow rates Re I, II, and III, respectively. Maximum velocity of the fluid is observed at the region of bifurcation and in internal branch of the right carotid artery in all the cases. Magnitude of maximum velocity is found conserved with the increased flow rates at the above-mentioned time steps. However, it is found that vortices get irregular in external branch and attenuated in internal branch with increased flow rates on due course of time. However, vortices vanished from internal branch of left carotid artery at highest flow rate (Re III). Vortices also appear in consistent manner in main branch of right carotid artery at upstream to stenoses.

Key markers in the form of gradient and harmonics of wall shear stress (WS) are used to address the above-mentioned investigations quantitatively. Fluid-mechanical parameters such as WSS, pressure contour, vorticity, and velocity-distributions are evaluated from time-dependent primitive variables. TAWSS [8] is calculated to measure distribution of WSS at luminal surface of blood vessel, and it is calculated by using

(9) $$\begin{array}{c} TAWSS=\frac{1}{T}\overset{T}{\underset{0}{\int }}\left|\;WSS\left(\;s,t\;\right)\;\right|dt. \end{array}$$

TAWSS is used for quantitative measurement of abnormality in flow structure. Low value of TAWSS (lower than 0.4 Pa) stimulates proatherogenic endothelial phenotype [19]. Perturbed endothelial-alignment on wall of vessel is induced at regions susceptible to prolong (large fraction of cardiac cycle) deviation of instantaneous WSS from direction of main flow.

**Figure 6:**
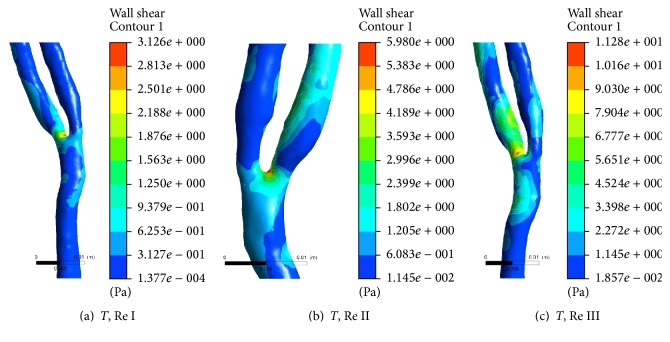
Contour plot of wall shear stress at bifurcated section of healthy carotid artery at the end of one complete cardiac cycle at Re 544, 1088, and 2176.

Figure 6 shows the variations of WSS in form of contour plot at bifurcated section of healthy carotid artery at the end of one complete cardiac cycle at different Re of 544, 1088, and 2176 while considering Power Law rheological model. It is observed that spatial distribution of WSS varies with flow rates. At low flow rate, region of highest WSS is confined at point of bifurcation. However, diffused distribution of WSS is also observed at further downstream in internal carotid artery on increased flow rates from Re I to Re III. It confirms the regions to be highly prone to wear and tear, which make the region favourable to secondary stenoses.

**Figure 7:**
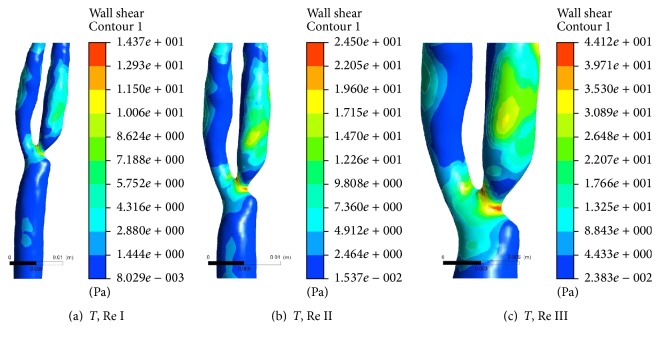
Contour plot of wall shear stress at bifurcated section of diseased carotid artery at the end of one complete cardiac cycle at Re 954, 1908, and 3816.

Figure 7 shows the variations of wall shear stress in the form of contour plot at bifurcated section of diseased carotid artery at the end of one complete cardiac cycle at different Re of 954, 1908, and 3816 while considering Power Law rheological model. In diseased vessel containing stenoses at the point of bifurcation, behaviour of WSS remains almost similar to that in healthy vessel with some additional recognizable features. Magnitude of WSS is found much higher in carotid artery with stenoses than that in healthy carotid artery. It is observed that confinement of high magnitude of WSS at region of stenoses even extended in upstream to stenoses. Simultaneously, distribution of elevated WSS is also observed in internal branch of carotid artery containing stenoses, in contrary to the response of the same branch to velocity streamline function. This secondary level of WSS further spread spatially to further downstream of bifurcation (particularly in internal carotid artery) on increased flow rates (from Re I to Re III). It also confirms the regions to be highly prone to wear and tear, making it favourable for secondary stenoses.

WSS-based descriptors are also used as markers for calculating various states of ECs of vessel. Gradient based descriptors and harmonic based descriptors are also calculated in this paper. WSSG is a marker of endothelial cell tension. It is calculated from WSS-gradient tensor components parallel and perpendicular to TAWSS vector (*m* and *n*, resp.) [20], and it is given by

(10) $$\begin{array}{c} WSSG=\frac{1}{T}\overset{T}{\underset{0}{\int }}\sqrt{{\left(\frac{\partial \tau_{w,m}}{\partial m}\right)}^{2}+{\left(\frac{\partial \tau_{w,n}}{\partial n}\right)}^{2}}dt. \end{array}$$

Table 3 represents transient evaluations of WSSG in diseased and healthy carotid artery at different Reynolds numbers. WSSG is found severalfold higher in diseased vessel than in healthy vessel at same Re and it holds true on increased flow rate too. However, magnitude of WSSG is also observed to increase with increased flow rate.

An interesting finding is observed about the behaviour of WSSG to different rheological models. Quemada model represents higher response to WSSG in comparison to Power Law model. Repetitive strong response of Quemada model to shear stress indicates that Quemada model may handle flow of higher shear rates at irregular geometries like highly asymmetric bifurcation channel.

**Figure 8:**
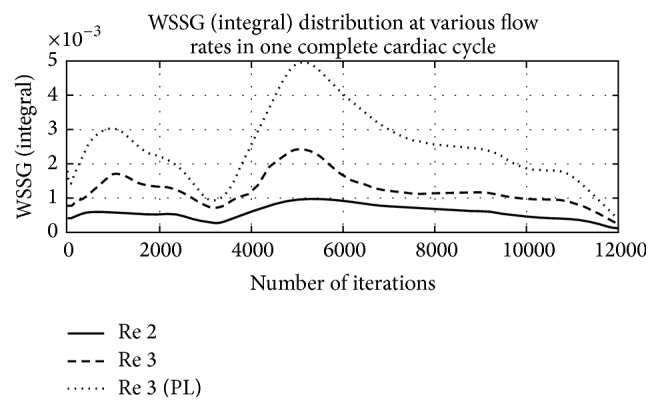
Graphical representation of WSSG distribution in healthy blood vessel at different Re.

Figure 8 shows a comparative behaviour of WSSG in a healthy vessel under the influence of variations in flow rates. It is observed that time integral form of WSSG shows greater response to Quemada model than Power Law model. Thus, again it confirms Quemada model to be a better rheological marker in asymmetric geometries.

Maximum absolute rate of change in magnitude of WSS over a cardiac cycle is also known as WSST, and it is calculated by

(11) $$\begin{array}{c} WSST=\max\left(\;\left|\;\frac{\partial \left|\tau_{w}\right|}{\partial t}\;\right|\;\right). \end{array}$$

Table 4 represents transient evaluations of WSST in diseased and healthy artery at Reynolds numbers Re I, Re II, and Re III, respectively.

Behaviour of WSST does not hold similar to that of WSSG for Quemada model. Here, Quemada model shows better responses to WSST, but it also depends on flow rate. It is observed that WSST decreases with increased flow rate in healthy vessel. However, an oscillatory behaviour of WSST is found in diseased vessel with increased flow rate.

**Figure 9:**
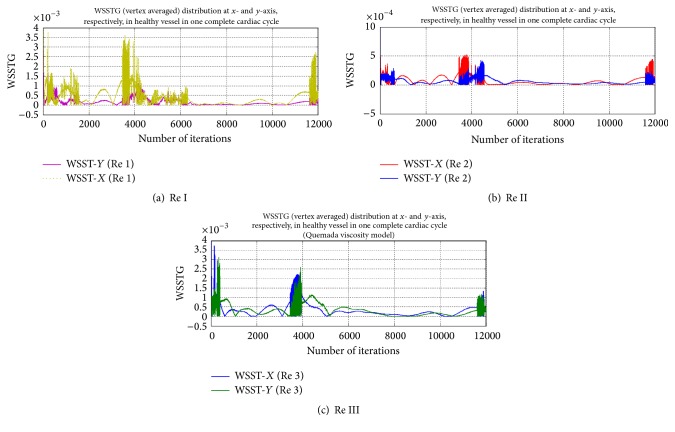
Graphical representation of WSST distribution in healthy blood vessel at different Re I, II, and III.

Figure 9 shows a comparative behaviour of WSST in a healthy vessel at different flow rates. WSST in axial direction (*x*-direction) is found more pronounced than WSST in radial direction (*y*-direction) at Re I and Re II. However, on increased flow rate (Re II), an oscillatory behaviour of WSST is observed in both *x* and *y* directions. WSST in radial direction is found greater than that in axial direction on increased flow rate to Re III.

**Figure 10:**
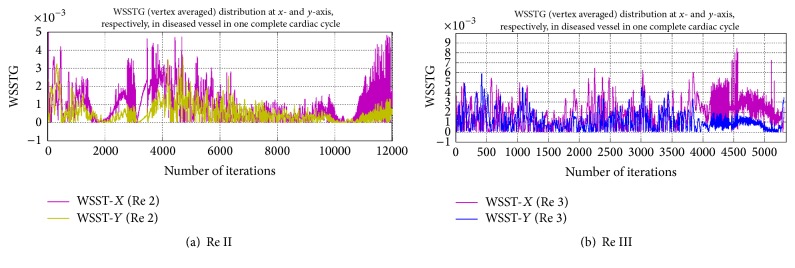
Graphical representation of WSST distribution in diseased blood vessel at different Re II and III.

Figure 10 shows a comparative behaviour of WSST in a diseased vessel under the influence of variations in flow rates.

It is observed that magnitude of WSST in axial direction is more than that in radial direction in both cases. However, the difference in axial WSST and radial WSST is minimum at the beginning of the cardiac cycle, and it increases with progression of the cardiac cycle.

Harmonic content of WSS waveforms is a possible metric of disturbed flow. This statement is enforced by results revealing that endothelial cells sense and respond to the frequency of WSS profiles. Time-varying magnitude of WSS at each node is Fourier-decomposed, and DH is defined as harmonic with the highest amplitude [21]. It is calculated by

(12) DH=max⁡Fwnω0,Fw≡FFTτw,   ω0=2πT.

Table 5 represents transient evaluations of DH in diseased and healthy artery at Reynolds numbers Re I, Re II, and Re III, respectively. Table shows that value of DH increases with increased flow rate in diseased vessel, whereas it behaves in oscillatory manner in case of healthy vessel. However, DH also shows better response to the flow while considering rheological property of the fluid as Quemada model.

## 4. Discussion

Flows at different Re in bifurcated section of healthy carotid artery under the influence of diseased state of another carotid branch are evaluated. Region of bifurcation is found to play important role as flow divider, which enhances the magnitude of flow. Vortices are formed in downstream of bifurcation due to division in flow at lower flow rate. However, vortex shedding occurs at increased flow rate due to gain in momentum of blood which dissociates larger eddies into smaller ones and thus releases kinetic energy associated with flow. However, viscosity is found to play less significantly in order to govern the pattern in vortex shedding in flow of blood in these vessels. Since both Power Law and Quemada viscosity models are non-Newtonian in nature, therefore, at lower strain rate, influence of these two non-Newtonian viscosity models vanishes due to Newtonian behaviour of blood at high strain rate. However, influences of Quemada model are found in this case even at high strain rate. It is due to the presence of more haematocrit and globules of thrombus in diseased blood due to presence of stenoses in the opposite branch of carotid artery.

Influences of stenoses on vortices at different flow rates are found in downstream of stenoses. Internal branch is found to have dissociation in vortices at higher Re due to increased rate of dissipating kinetic energy from larger eddies, whereas irregularities in structure of vertices in outer branch are due to gain in angular momentum at high Re.

Variations of WSS are also observed at different flow rates in healthy branch of the carotid artery. Regions with bifurcation disturbed the downstream flow of diseased blood which may lead to larger contact areas between the complex of haematocrit and thrombus with endothelial wall of vessels. And on increased flow rate, intensity of contact increases, which leads to increased shear rate at the surface of wall, making the region favourable for secondary stenoses. Since stenoses is found at internal side of bifurcation in the opposite carotid artery, therefore, its influence on the healthy branch is also found greater in the mirror-artery which may be due to conservation law for fractal nature of the branches of vessels of carotid arteries. Presence of stenoses in RCA makes the internal RCA a favourable place for secondary stenoses, because highest WSS distribution is found in downstream of stenoses in this branch even at higher flow rate in comparison to external branch of RCA and internal branch of LCA. Such observation leads to more intense qualitative measurement of WSS-based descriptors on endothelial cell linings of the wall of both RCA and LCA.

Gradient based descriptors of WSS represent the effect of rate of change in WSS on ECs, whereas harmonic based descriptors decompose the cumulative effects of WSS on ECs into individual vector components in order to represent the effect of WSS on ECs with respect to directions, magnitude, and phase. Since gradient operator takes into account both parallel and perpendicular components of WSS, thus response of both normal and shear stress on ECs can be understood in better way.

In diseased vessel, anomalies are associated with the ECs of wall of vessels. And it is very well justified from the pattern of WSSG as tabulated in Table 3. Also, Quemada model is found to respond to evaluation of WSSG than that by Power Law model; it is because the haematocrit components of blood are best modelled by Quemada model, particularly in diseased condition of vessel. The same phenomenon was also found true by Bit and Chattopadhyay [22] in a stenoses containing straight blood vessel.

However, WSST is found to be flow-driven parameter because the function itself is a transient function. Therefore, variation in flow rates also governs the variation of WSST independent of viscosity model used at a particular pathophysiological state of blood (i.e., either in diseased condition or in healthy condition). Since the flow of blood in vessel is in axial direction, value of WSST is much more in *x*-direction in comparison to that in *y*-direction at low and normal flow rate. However, at higher flow rate, oscillatory behaviour of WSST may be due to development of chaos in flow structure, which might have occurred due to random interaction of RBCs and microthrombus of plaque in diseased vessel, such kind of interactions makes the flow domain unstable in direction of flow, and it leads to significant flow component in direction perpendicular to flow direction. Harmonic components of WSS give the measure of cellular responses and tendency to fatigue of ECs. Each time a component of harmonic interacts with ECs in spatiotemporal domain, cells respond accordingly in a bidirectional format (i.e., cellular responses to WSS, and a messenger response to deeper layer of cells for neurovascular activities). Response time and repeatability are critical factors for ECs, and it becomes further complicated in diseased condition of ECs. Therefore, measure of harmonic content of WSS at downstream regions of stenoses will give a measure of identification of spatial regions where endothelial cell lyses or irregular ECs response to the flow of blood is likely to happen.

Therefore, based on the measurement done by these useful WSS markers, the regions prone to secondary stenoses in carotid artery with existing stenoses are confirmed. It is observed that regions of internal carotid artery of LCA are prone to secondary blockage due to the influence of primary blockage in RCA. However, regions of both internal and external branches of RCA are found prone towards secondary stenoses. It is also observed that higher metabolic rate can reduce chances of secondary stenoses in both bifurcated arteries. Simultaneously, gradient operator and harmonic operator of WSS are found to be effective tools for determining region of secondary stenoses, particularly with respect to temporal variations in WSS, over a cardiac cycle. Therefore, gradient based and harmonic based markers of WSS are found useful tools for detection of spontaneous triggering of secondary stenoses in downstream of primary stenoses at well-advanced stage when the influence of primary blockage is started on endothelial cell linings.

## Figures and Tables

**Table 1:** Mesh element statistics of diseased and healthy vessels.

Carotid artery	Types of mesh element	Total number of elements	Total area mm^2^	Total volume mm^3^	Inlet diameter (mm)	ECA diameter (mm)	ICA diameter (mm)	Height (mm)
Right CA (diseased)	Tetrahedral: 178808;	312615	3438.76	4921.87	9.09	2.39	2.73	161.75
	Triangular: 22516;							
	Pentagonal: 99903;							
	Quadrilateral: 291							

Left CA (healthy)	Tetrahedral: 178968;	211642	2942.26	3293.18	5.02	3.23	3.59	156.91
	Triangular: 32590							

**Table 2:** Different flow rates of respective carotid arteries.

Carotid artery (CA)	Mean flow rate (m/sec)	Re I	Re II	Re III
Right CA (RCA), diseased	0.15	954	1908	3816
Left CA (LCA), healthy	0.15	544	1088	2176

**Table 3:** WSSG in both diseased and healthy vessels.

Viscosity model	Flow rate	Healthy vessel	Diseased vessel
		WSSG	WSSG
Power Law	Re 1	2.8512	32.7799
	Re 2	7.1872	73.9769
	Re 3	15.1477	89.4231

Quemada	Re 3	30.4866	115.8264

**Table 4:** Transient form of WSST in both diseased and healthy vessels.

Viscosity model	Flow rate	Healthy vessel	Diseased vessel
		WSST	WSST
Power Law	Re 1	0.0147	0.0147
	Re 2	0.0127	0.0187
	Re 3	0.0094	0.0085

Quemada	Re 3	0.0122	0.0101

**Table 5:** DH in both diseased and healthy vessels.

Viscosity model	Flow rate	Healthy vessel	Diseased vessel
		DH	DH
Power Law	Re I	30.6964	30.6964
	Re II	5.5557	70.6169
	Re III	13.2493	82.7616

Quemada	Re III	24.0394	84.4652

## Abbreviations

CA: Carotid artery
LCA: Left carotid artery
RCA: Right carotid artery
WSS: Wall shear stress
EC: Endothelial cell
DH: Dominant harmonic
ICA: Internal carotid artery
CT: Computerised tomography
MRI: Magnetic resonance imaging
TAWSS: Time averaged wall shear stress
OSI: Oscillatory shear index
WSST: Temporal gradient of WSS.

### Symbols

*p*: Absolute pressure
*u*: Axial velocity
*ρ*: Density of blood
*v*: Radial velocity
*ω*: Angular velocity
*y*: *y*-Coordinate
Re: Reynolds number
*u*(*t*): Transient velocity along axis of tube
*r*: Characteristics of radial length
*μ*_app_: Apparent viscosity of blood
*x*: Characteristics of length of vessel
*ε*_rms_: Root-mean-square error
*x*[*i*, *j*]: Position-vector of control-volume
*h*: Inlet diameter of vessel.

## Additional Points

Computerised scanned data of upper thorax regions were obtained from patient suffering from the respective disease of interest. However, no additional instruction was provided to the patient, and no additional task was performed beyond routine examination to the patient for the purpose of data collection only. Data was collected from hospital database, keeping the identity of the subject entirely masked. Therefore, the study is not directly related to human study, as no products developed from the study are directly tested on human subject.
